# Targeted Long-Read Sequencing Decodes the Transcriptional Atlas of the Founding RAS Gene Family Members

**DOI:** 10.3390/ijms222413298

**Published:** 2021-12-10

**Authors:** Panagiotis G. Adamopoulos, Panagiotis Tsiakanikas, Michaela A. Boti, Andreas Scorilas

**Affiliations:** Department of Biochemistry and Molecular Biology, Faculty of Biology, National and Kapodistrian University of Athens, 15701 Athens, Greece; padamopoulos@biol.uoa.gr (P.G.A.); ptsiak@biol.uoa.gr (P.T.); miboti@biol.uoa.gr (M.A.B.)

**Keywords:** alternative splicing, nanopore sequencing, long-read sequencing, transcriptome, GTPases

## Abstract

The complicity of human RAS proteins in cancer is a well-documented fact, both due to the mutational hyperactivation of these GTPases and the overexpression of the genes encoding these proteins. Thus, it can be easily assumed that the study of *RAS* genes at the transcriptional and post-transcriptional level is of the utmost importance. Although previous research has shed some light on the basic mechanisms by which GTPases are involved in tumorigenesis, limited information is known regarding the transcriptional profile of the genes encoding these proteins. The present study highlights for the first time the wide spectrum of the mRNAs generated by the three most significant *RAS* genes (*KRAS*, *NRAS* and *HRAS*), providing an in-depth analysis of the splicing events and exon/intron boundaries. The implementation of a versatile, targeted nanopore-sequencing approach led to the identification of 39 novel *RAS* mRNA transcript variants and to the elucidation of their expression profiles in a broad panel of human cell lines. Although the present work unveiled multiple hidden aspects of the *RAS* gene family, further study is required to unravel the biological function of all the novel alternative transcript variants, as well as the putative protein isoforms.

## 1. Introduction

The human RAS superfamily of small guanosine triphosphatases (GTPases) consists of more than 150 members and is subdivided into five distinct categories (RAS, RHO, RAB, ARF and RAN) on the basis of the protein-members’ sequences, structures and functional characteristics [1]. The RAS subfamily consists of GTPases that are anchored to the membrane via post-transcriptional modifications that occurred at their C-terminal region, guiding their localization into distinct membrane compartments [2,3,4] where they are capable of regulating signal-transduction pathways [5]. In specific, the RAS GTPases KRAS, NRAS and HRAS represent the founding members of this large superfamily of monomeric small (20–25 kDa) GTPases, which play a major role in the regulation of fundamental cellular processes, including cell-cycle progression, cell survival, cell polarity and movement, actin cytoskeletal organization as well as vesicular and nuclear transport [6,7].

According to the GenBank^®^ database, the human genes that encode the founding Ras GTPases (*KRAS*, *NRAS* and *HRAS*) produce 9 protein-coding alternative splice variants that lead to the generation of 6 distinct protein isoforms. These isoforms are characterized by high sequence similarities, sharing major structural and functional features. In particular, the different RAS isoforms share their highest similarity in the N-terminal, where the catalytic domain (also known as G-domain) is located. The G-domain consists of the residues 1–166 and propagates signaling when bound to GTP, whereas it is inactive when bound to GDP. Specifically, it consists of two lobes and its active site resides within the first half of the domain, referred as the effector lobe (residues 1–86). The specific part of the catalytic domain contains three of the five highly conserved motifs that are observed in small GTPases [8,9], and is 100% identical in sequence among the RAS isoforms [10,11]. The second half of the catalytic domain constitutes the allosteric lobe (residues 87–166), which is characterized by the presence of two motifs, NKXD and EXSAK. The NKXD motif (residues 116–119) is responsible for the specificity of the guanine nucleotide [12], while the EXSAK motif (residues 143–147) subserves the stabilization of the nucleotide binding. The allosteric lobe contains hot spots of protein–ligand interactions that bind membrane components and harbors all the differences between the RAS G-domains at the level of primary structure, with a sequence identity of 90% among the allosteric lobes of the three RAS proteins [11,13,14]. Of note, the G-domain harbors both switch I (residues 30–40) and II (residues 60–76) (S1 and S2) regions, as well as the P-loop (residues 10–17) [15], which are responsible for mediating GTP hydrolysis [5,16] and altering the conformational status of RAS isoforms during the GDP/GTP exchange cycle [17,18], while forming an interaction surface for different effectors and regulators [5].

Their biological role as binary molecular switches places them among the key signal transducers, controlling cellular growth, differentiation, survival and motility as well as cytoskeletal plasticity. More precisely, multiple extracellular stimuli modify the dynamic equilibrium between the inactive (GDP-bound) and active (GTP-bound) states of RAS proteins. As a result, activated RAS-GTP complexes accumulate intracellularly and interact with several downstream effectors to activate critical signaling cascades [7,19]. Because of their involvement in a such significant pathway, these proteins unsurprisingly undergo strict regulation. In particular, RAS GTPases exhibit high-affinity binding for GDP and GTP, but possess low intrinsic GTP-hydrolysis and GDP/GTP-exchange activities [7], which are regulated by two major classes of regulatory proteins. Guanine-nucleotide-exchange factors (GEFs) mediate the composition of an active GTP-bound form [20], whereas GTPase-activating proteins (GAPs) accelerate the intrinsic GTPase activity to mediate the formation of the inactive GDP-bound form [21].

A well-documented fact in the existing literature is the high prevalence of RAS mutations across human malignancies. The major representatives of the *RAS* gene family, namely *HRAS*, *KRAS* and *NRAS,* are the most frequently mutated genes in malignant neoplasms, and have been under the spotlight of scientific research for the development of targeted therapies [22]. The majority of these mutations contribute to the self-sustainability of the active RAS-GTP state by evading the regulatory circuit of the guanine-exchange cycle, in which GAPs and GEFs tightly regulate GDP/GTP exchange between different RAS states [23,24]. Since these signals are firmly associated with cell growth and division, overactivated RAS signaling can ultimately lead to carcinogenesis [25,26,27,28,29]. Recent scientific evidence shows that among the RAS isoforms, missense mutations are predominantly found in *KRAS* (85%) and much less frequently in *NRAS* (12%) and *HRAS* (3%) [30]. In fact, the founding members of the *RAS* gene family in humans (*KRAS*, *NRAS* and *HRAS*) are the most common oncogenes in human cancer, whereas mutations that permanently activate RAS are found in almost 25% of all human tumours and up to 90% in specific types of malignancies [31,32,33,34,35,36]. For this reason, RAS inhibitors have been the main subject of research into novel therapeutic strategies in human cancers that are characterized by RAS overactivation.

Apart from their mutational activation, the altered expression patterns of *RAS* genes have been found in several human malignancies [37]. For example, two of the *KRAS* alternative splice variants, namely *KRAS-4A* and *KRAS-4B*, have been found to encode oncogenic proteins when *KRAS* is mutated [38]. Although the current evidence for their differential functions is limited, mutated *KRAS-4A* and *KRAS-4B* differ in their ability to induce anchorage-independent growth and cell migration. Furthermore, studies in animal models have indicated that wild-type *KRAS-4A* has tumour-suppressive and pro-apoptotic activity, whereas the wild-type *KRAS-4B* can be characterized as anti-apoptotic [39,40]. In addition, recent reports have confirmed that the expression levels of the *KRAS-4A* transcript are notably decreased in colorectal adenocarcinoma (CRC), highlighting that *KRAS* splicing is notably altered and therefore indicating that the balance among the expression levels of the *KRAS* splice variants may have a significant role in CRC tumorigenesis [41]. In contrast to their mutational status, which has been thoroughly investigated, the transcriptional landscape of the *RAS* gene family and the effect of the alternative splicing mechanism in this superfamily remain unclear. As a result, the thorough investigation of *RAS* pre-mRNA processing and the identification of the actual mature mRNAs that are synthesized under normal and/or pathological conditions are of high significance, as they may lead to the identification of new diagnostic and/or prognostic biomarkers or promising novel therapeutic targets.

To this end, the present study aims to identify new mRNA transcripts of the founding members of the RAS gene family using the cutting-edge nanopore-sequencing technology. However, instead of using the “gold-standard” direct-RNA-sequencing application, which fails to identify novel transcripts due to the seriously decreased sequencing depth in terms of a specific gene, we designed and employed a targeted nanopore-sequencing approach that offered a tremendous sequencing depth, which allowed the thorough investigation of the existing alternative splicing events and the identification of novel *RAS* mRNA transcripts.

## 2. Results

### 2.1. Nanopore Sequencing Reveals Novel RAS mRNA Transcripts

**Figure 1:**
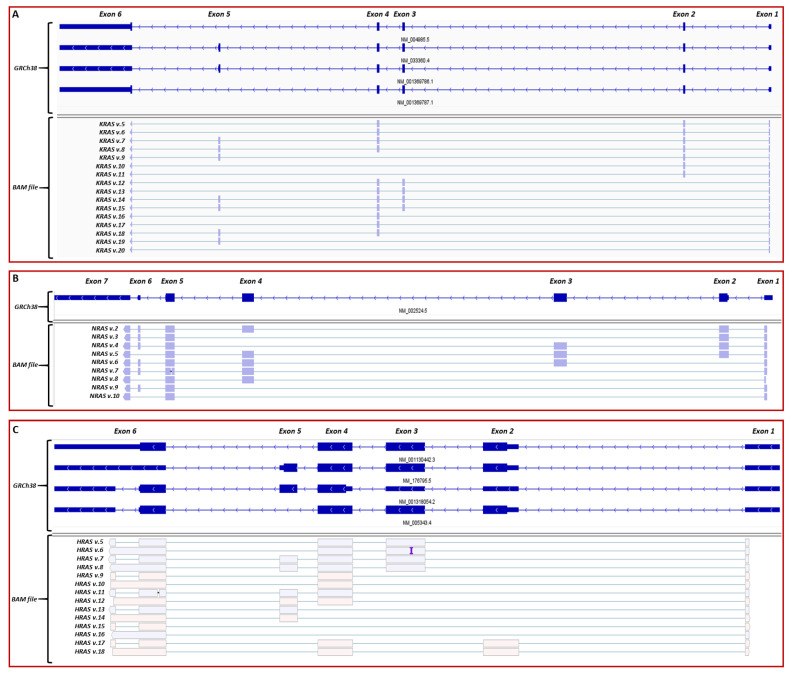
Visualization of aligned sequencing reads representing the novel *RAS* mRNA transcripts described in the present study, using IGV. The human genome 38 (GRCh38) was used as reference. (**A**) Visualization of the 16 novel *KRAS* transcripts (*KRAS* v.5 – v.20). (**B**) Visualization of the 9 novel *NRAS* transcripts (*NRAS* v.2 – v.10). (**C**) Visualization of the 14 novel *HRAS* transcripts (*HRAS* v.5 – v.18).

Visualization of the aligned nanopore-sequencing reads with Integrative Genomics Viewer (IGV) confirmed the annotated mRNA transcripts of *KRAS*, *NRAS* and *HRAS* genes with significant depth. However, a bioinformatics analysis also unveiled novel splice junctions between annotated exons of these genes, which were confirmed by our *in-house* developed generic splicing tool “ASDT” [42] and visualization of the mapped sequencing reads with IGV (Figure 1). Notably, due to the long-read technology that nanopore sequencing offers, the derived sequencing reads that bear the novel splice junctions covered the entire cDNA sequences from the first until the last exon of each *RAS* gene, thus representing novel *RAS* mRNA transcripts (Supplementary data). In addition, it should be mentioned that even though the frequency of each new splicing event was decreased as compared to the annotated ones, each newly identified *RAS* mRNA transcript was validated by multiple nanopore-sequencing reads. For all three of the *RAS* genes that were investigated in the present work, a total of 39 novel mRNA transcripts were identified and their exon/intron boundaries were fully characterized (Figure 1).

### 2.2. Identification of Novel KRAS mRNA Transcripts

**Figure 2:**
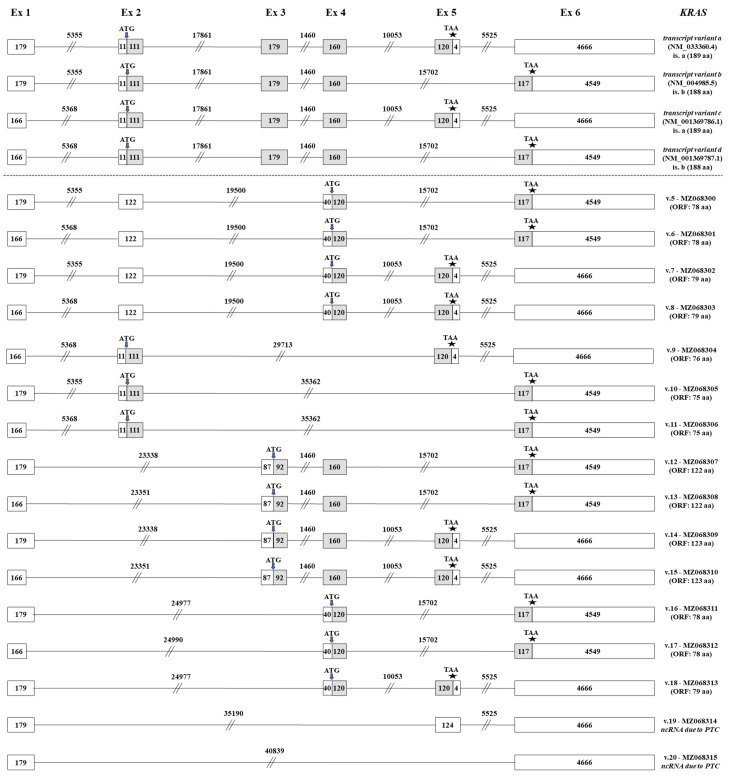
Detailed structural demonstration of the 16 novel *KRAS* transcript variants (*KRAS* v.5 – v.20) identified in the current study. Exons are exhibited as boxes and introns as lines, while the numbers that characterize every box and line correspond to their length in nucleotides. Gray and white boxes represent coding and non-coding regions, respectively. The positions of the ATG and stop codons are pointed out with arrows (↓) and asterisks (*), accordingly.

Briefly, 4 annotated *KRAS* transcript variants (variants a, b, c and d, GenBank^®^ accession numbers: NM_033360.4, NM_004985.5, NM_001369786.1 and NM_001369787.1, respectively), which were generated by a total of six distinct exons, have already been confirmed (Figure 2). Variants a and c encode the main KRAS isoform (189 aa), while variants b and d synthesize an alternative KRAS isoform of 188 aa. The two transcripts that encode each isoform are almost identical, since they only contain a subtle differentiation in their first exon, which is characterized by an alternative 5’ splice site. Therefore, the first exon of variants a and b is composed of 179 nt, whereas variants b and d encompass a truncated exon 1 (166 nt). Besides the validation of these 4 annotated *KRAS* transcript variants, the sequencing results of the current study confirmed the existence of 16 novel *KRAS* mRNA transcripts (*KRAS* v.5 – v.20, GenBank^®^ accession numbers: MZ068300–MZ068315, accordingly) that are characterized by new exon-skipping events.

Based on the obtained raw reads, 4 new *KRAS* transcript variants (*KRAS* v.5 – v.8) share the novel splicing event between exons 2 and 4, thus lacking the sequence of exon 3 (Figure 2). Although *KRAS* v.5 and v.6 are also characterized by the exon skipping of exon 5 that is observed on two annotated variants (b and d), this exon is present on both *KRAS* v.7 and v.8. In addition, *KRAS* v.5 and v.7 encompass the annotated first exon that consists of 179 nt, while *KRAS* v.6 and v.8 bear the alternative truncated exon 1 (166 nt). This subtle difference in the first exon is also encountered on the rest of the identified *KRAS* transcripts, leading to pairs of new variants that contain only this subtle differentiation. An *in silico* query for open reading frames (ORFs) indicated that *KRAS* v.5 – v.8 are predicted to encode new KRAS isoforms, utilizing an alternative translation-initiation codon that resides in exon 4 (Figure 2).

An additional *KRAS* mRNA transcript was identified (*KRAS* v.9), which is characterized by the truncated exon 1 (166 nt) and the simultaneous absence of exons 3 and 4 from its cDNA sequence. Despite this new splicing event between exons 2 and 5, *KRAS* v.9 is predicted to be protein-coding since it has an ORF of 76aa (Figure 2). The next two novel transcripts, *KRAS* v.10 and v.11, share the previously undescribed splicing event between exons 2 and 6, while being differentiated only in the 5’ splice site of the first exon. Both *KRAS* v.10 and v.11 have ORFs, utilizing annotated start and stop codons and, therefore, are predicted to be protein-coding mRNAs.

Furthermore, our findings confirmed a set of 4 novel *KRAS* mRNA transcripts (*KRAS* v.12 – v.15), which share the exon skipping of exon 2, an exon that is present in all the annotated *KRAS* transcripts. Both *KRAS* v.12 and v.13 are also characterized by the exon skipping of exon 5, while *KRAS* v.14 and v.15 contain the entire exon 5 sequence. Once again, these two pairs of transcripts are further differentiated based on whether they encompass the truncated exon 1 (Figure 2). Regarding their protein-coding ability, *KRAS* v.12 – v.15 are expected to be protein-coding, utilizing an alternative translation-initiation codon residing on exon 3.

Besides the previously described findings, the derived sequencing reads led to the identification of 3 additional novel *KRAS* transcripts (*KRAS* v.16 – v.18) that are characterized by the new splicing event between exons 1 and 4. These splice variants have notably truncated nucleotide sequences as compared to the annotated ones, since both exons 2 and 3 are absent. Two of these transcripts, *KRAS* v.16 and v.17, also lack exon 5 and are differentiated in the 5’ splice site of the first exon. On the contrary, *KRAS* v.18 contains exon 5 and encompasses the full sequence of exon 1 (179 nt). This new splicing event between exons 1 and 4 leads to the creation of ORFs and therefore *KRAS* v.16 – v.18 are most likely protein-coding mRNAs. Finally, our findings unveiled two non-coding mRNA transcripts, *KRAS* v.19 – v.20, which are significantly truncated transcripts as they are generated by new splicing events between distant exons (Figure 2).

### 2.3. Identification of Novel NRAS mRNA Transcripts

Only a single annotated transcript (*NRAS* v.1, GenBank^®^ accession number: NM_002524.5) is currently curated for the human *NRAS* gene. This mRNA is composed of 7 distinct exons and encodes the NRAS GTPase. Despite *NRAS* v.1 being the most abundant mRNA in the obtained sequencing data, a computational analysis confirmed multiple novel exon-skipping events that lead to the generation of 9 novel *NRAS* mRNA transcripts (*NRAS* v.2 – v.10, GenBank^®^ accession numbers: MZ068316–MZ068324, respectively).

**Figure 3:**
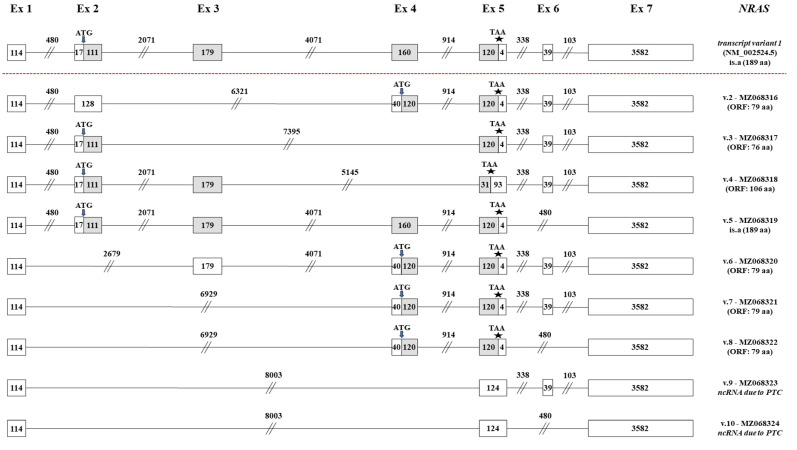
Detailed structural demonstration of the 9 novel *NRAS* transcript variants (*NRAS* v.2 – v.10) identified in the current study. Exons are exhibited as boxes and introns as lines, while the numbers that characterize every box and line correspond to their length in nucleotides. Gray and white boxes represent coding and non-coding regions, respectively. The positions of the ATG and stop codons are pointed out with arrows (↓) and asterisks (*), accordingly.

A bioinformatics analysis unveiled a set of 5 *NRAS* transcripts (*NRAS* v.2 – v.6), each one derived from a new exon-skipping event. In detail, *NRAS* v.2 is produced by the exon skipping of exon 3, whereas *NRAS* v.3 lacks both exons 3 and 4 (Figure 3). Additionally, *NRAS* v.4 is characterized by the exon skipping of exon 4 and *NRAS* v.5 is produced by the new splicing event between exons 5 and 7, thus lacking exon 6. Finally, *NRAS* v.6 lacks exon 2, which contains the annotated translation-initiation codon. Although *NRAS* v.2 – v.6 are derived from different exon-skipping events, they all have ORFs and as a result they most likely represent protein-coding mRNAs (Figure 3).

Furthermore, our findings indicate the existence of two additional splice variants, *NRAS* v.7 and v.8. These two novel *NRAS* transcripts share the new splicing event between exons 1 and 4 but are differentiated since *NRAS* v.8 also lacks exon 6. The simultaneous exon skipping of exons 2 and 3 in these transcripts leads to the creation of the same ORF, and therefore *NRAS* v.7 and v.8 are predicted to encode the same protein isoform. In addition, the last two identified transcripts, *NRAS* v.9 and v.10, share the new splicing event between exons 1 and 5, thus being significantly truncated as compared to the annotated *NRAS* v.1. Their difference is detected on exon 5, which is present on *NRAS* v.9, but absent from *NRAS* v.10 (Figure 3). Finally, the new splicing of exons 1 and 5 leads to the formation of a premature termination codon (PTC) and, as a result, both *NRAS* v.9 and v.10 are highly expected to represent non-coding mRNAs.

### 2.4. Identification of Novel HRAS mRNA Transcripts

**Figure 4:**
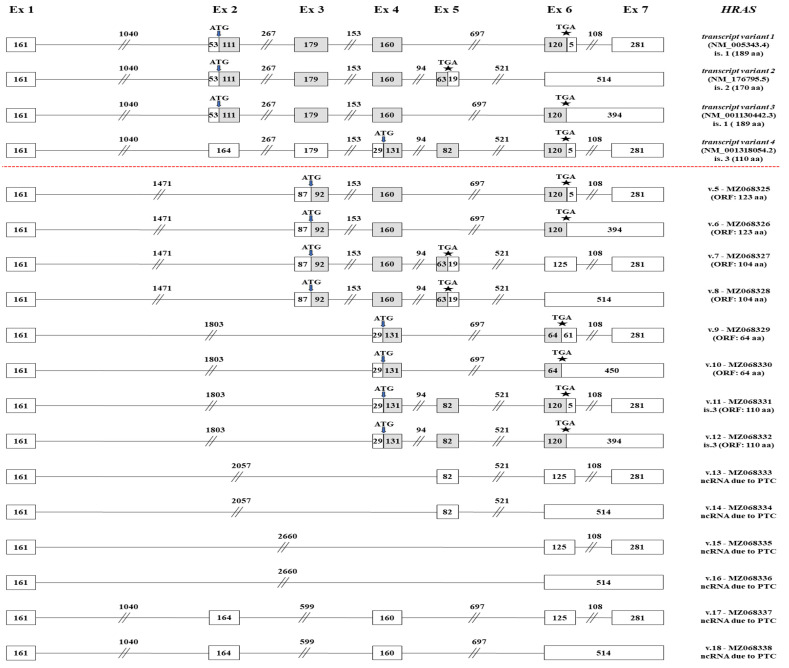
Detailed structural demonstration of the 14 novel *HRAS* transcript variants (*HRAS* v.5 – v.18) identified in the current study. Exons are exhibited as boxes and introns as lines, while the numbers that characterize every box and line correspond to their length in nucleotides. Gray and white boxes represent coding and non-coding regions, respectively. The positions of the ATG and stop codons are pointed out with arrows (↓) and asterisks (*), accordingly.

Based on the existing literature, 4 annotated *HRAS* transcripts have been already confirmed (*HRAS* v.1 – v.4, GenBank^®^ accession numbers: NM_005343.4, NM_176795.5, NM_001130442.3 and NM_001318054.2). In total, 7 distinct exons of the gene have been elucidated, whereas an intron retention between exons 6 and 7 has also been identified in *HRAS* v.2 and v.3 (Figure 4). Although our nanopore sequencing results validated all the annotated mRNAs, they also revealed the existence of 5 new alternative splicing events that eventually led to the identification of 14 novel *HRAS* mRNA transcripts (*HRAS* v.5 – v.18, GenBank^®^ accession numbers: MZ068325–MZ068338, accordingly).

Initially, we identified a set of 4 novel *HRAS* mRNA transcripts (*HRAS* v.5 – v.8), which share the previously unidentified exon skipping of exon 2, since exon 1 is alternatively spliced with exon 3 (Figure 4). Besides this new splicing event, both *HRAS* v.5 and v.6 lack the complete sequence of exon 5 and are differentiated in the intron retention between exons 6 and 7, which is present only on *HRAS* v.6. On the contrary, both *HRAS* v.7 and v.8 encompass exon 5 and once again their difference is the intron retention between exons 6 and 7. Based on the in silico ORF query, *HRAS* v.5 – v.8 have ORFs and are therefore predicted to be protein-coding mRNAs. An additional set of 4 novel *HRAS* mRNA transcripts (*HRAS* v.9 – v.12) that share the new splicing event between exons 1 and 4 was unveiled (Figure 4). Besides the simultaneous absence of exons 2 and 3, these transcripts are differentiated based on whether they contain exon 5 and the intron retention between exons 6 and 7, which is a pattern that was also observed in the previously described set of transcripts (*HRAS* v.5 – v.8). Additionally, it should also be mentioned that *HRAS* v.9 – v.12 have ORFs, utilizing the annotated translation-initiation codon of *HRAS* v.4, and therefore they are expected to encode novel isoforms (Figure 4).

Finally, 6 additional *HRAS* mRNA transcripts with PTCs were identified and thus predicted to be non-coding mRNAs. These mRNAs can be divided into three pairs of transcripts, with each pair being differentiated only in the intron retention between exons 6 and 7. Briefly, *HRAS* v.13 and v.14 share the new splicing event between exons 1 and 5, while *HRAS* v.15 and v.16 are further truncated mRNAs as they are characterized by the new splicing event between exons 1 and 6 (Figure 4). The last two novel transcripts, *HRAS* v.17 and v.18, share the simultaneous exon skipping of exons 3 and 5.

### 2.5. Expression Pattern of the Novel RAS Transcripts in a Wide Spectrum of Human Tissues

**Figure 5:**
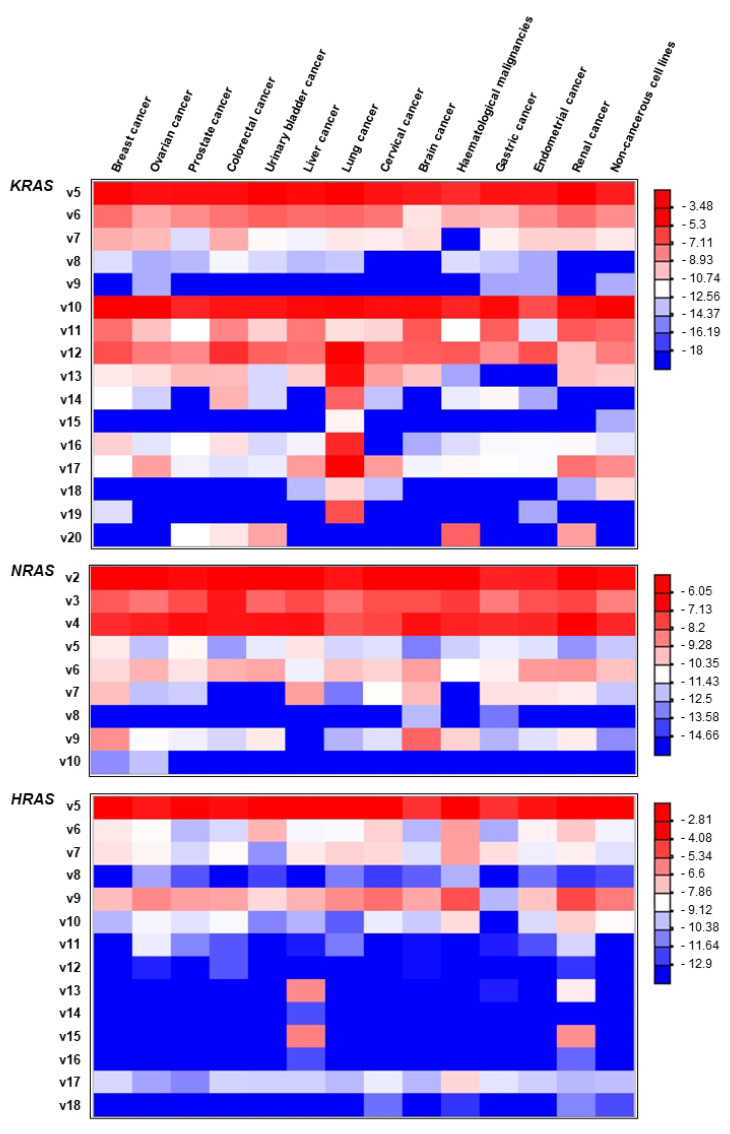
Heatmap demonstrating the fold change (Log2FC) ratios of each novel transcript variant relative to the abundance of the annotated mRNA(s) for each RAS gene. Negative values in the color scale bar indicate that all the novel alternative transcript variants are downregulated compared to the main transcript variant. Red coloring is indicative of higher relative abundance of the corresponding transcript variant, whereas blue coloring corresponds to low-frequent or undetectable transcript variants.

Although most of the novel transcript variants are detected in notably lower counts compared to the annotated mRNAs, interestingly, some of them demonstrate broad expression patterns across human tissues (Figure 5). In detail, the new transcript variants *KRAS* v5, v6, v10 and v12, *NRAS* v2, v3, v4 and *HRAS* v5 were identified as the most abundant in the human cell lines that were tested. On the contrary, *KRAS* v9, v15, and v19, *NRAS* v8 and v10 and *HRAS* v11, v12, v13, v14, v15, v16 and v18 were almost undetectable. However, some of the latter demonstrate a notable, tissue-specific expression. Specifically, *KRAS* transcript variants v13, v14, v15, v16, v17 and v19 were found to be highly expressed in lung cancer (Figure 5). Accordingly, based on our sequencing results, *NRAS* v9 is predominantly expressed in breast and brain cancer. Finally, *HRAS* v13 and v15 were notably expressed only in liver and renal carcinomas. This evidence supports a putative tissue-specific role of the corresponding transcript variants that merits further investigation regarding their biological role and their clinical value in human malignancies.

## 3. Discussion

In the present study, we developed and employed a versatile targeted nanopore sequencing approach to the in-depth detection of new alternative splicing events of the founding *RAS* family gene members (*KRAS*, *NRAS* and *HRAS*), and more specifically the identification of novel mRNA transcripts. The obtained sequencing data led to the identification of 39 novel *RAS* mRNA transcripts that originate from previously uncharacterized alternative splicing junctions between the annotated exons of these genes. Even though our research was mainly focused on the elucidation of novel splicing events occurring during RNA processing, the identification of the presented *RAS* transcripts was achieved by applying a targeted DNA-sequencing approach that exploited the nanopore-sequencing technology, instead of using the “gold-standard” direct-RNA-sequencing application [43]. The implementation of the presented targeted-sequencing approach offered a tremendous sequencing depth, enabling the identification and characterization of previously undetected mRNA transcripts in single reads. Of note, more conventional techniques for transcriptional analysis, such as direct-RNA, direct-cDNA or PCR-cDNA sequencing approaches are unable to detect novel transcript variants due to the significantly decreased sequencing depth they provide in terms of a specific gene.

Since RAS proteins play a crucial role in cancer, both due to the mutational hyperactivation of these GTPases and the overexpression of the genes encoding these proteins [22,23,24,37], it can be easily assumed that the study of these genes at the transcriptional and post-transcriptional level is of the utmost importance. The presumptive existence of transcript variants that have not yet been identified, as well as their potential association with specific types of cancers, may lay the foundations for the identification of novel diagnostic, prognostic and/or predictor biomarkers, and may also ameliorate the therapeutic management of cancers that are related to the oncogenic *RAS* genes [22,34].

Regarding the presented 16 novel alternative variants of *KRAS*, 14 of them are predicted to have ORFs and, thus, encode novel protein isoforms, whereas the remaining 2 transcripts contain PTCs and are therefore candidates for non-sense-mediated mRNA decay (NMD). Particularly, *KRAS* v.5 – v.11 and *KRAS* v.16 – v.18 are predicted to contain previously undescribed small ORFs (smORFs) and their potential translation would lead to the generation of KRAS micropeptides containing less than 100 aa [44]. Although the coding potential of these smORFs had not been elucidated in the past, recent studies have highlighted the crucial regulatory function of these small proteins [45,46,47]. Even though these studies provide new insights regarding the emerging role of these biomolecules in cellular physiology and widen our knowledge about the potential involvement of these molecules in human diseases, there is an urgent necessity for their further investigation to fully characterize and unveil their function in human malignancies. Regarding the structure of these KRAS micropeptides, the potential translation of *KRAS* v.5 – v.8 and v.16 – v.18 would lead to the production of small proteins that are expected to lack the effector lobe of the G-domain and a significant part of the allosteric lobe [11,48]. Although the residues that contribute to the formation of the allosteric lobe are present in the amino acid sequence of the aforementioned protein isoforms, the absence of the switch I and II regions indicate that these potential proteins are not capable of interacting with their downstream effectors [11,12,48]. On the contrary, *KRAS* v.9 – v.11 are predicted to encode KRAS micropeptides that are characterized by a truncated effector lobe, while they lack the allosteric lobe and as a result, they are not expected to mediate signaling. However, their C-terminal region is integral, enabling the transport of the particular proteins to the suitable membrane compartment [3,4].

The rest of the *KRAS* transcripts that are predicted to be coding (*KRAS* v.12 – v.15) are characterized by the presence of a novel translation-initiation codon. Specifically, *KRAS* v.12 and v.13 are predicted to encode the same protein isoform that consists of 122 aa, while the potential translation of *KRAS* v.14 and v.15 is expected to lead to the generation of a new protein that consists of 123 aa (Figure 2). These two novel protein isoforms are characterized by a truncated G-domain and a lack of the effector lobe, but retaining the allosteric lobe. Since the active site of the G-domain is missing, these isoforms would not be capable of mediating signaling [7]. However, the existence of a C-terminal that is identical to the C-terminal of the annotated protein isoforms indicates that these proteins can be transferred to the proper membrane compartment [3,4].

Besides the novel findings for *KRAS*, the in silico computational analysis led to the identification of 14 novel *HRAS* transcripts, 8 of which are predicted to encode new protein isoforms, while the rest of them contain PTCs, thus being NMD candidates. In specific, *HRAS* v.5 and v.6 are predicted to encode the same protein isoform, which is composed of 123 aa (Figure 4). This protein lacks a significant part of the effector lobe, including the switch I and II regions, and thus the specific isoform is not expected to interact with GAPs, which mediate GTP hydrolysis and alter the conformation of RAS isoforms during the GDP/GTP-exchange cycle [21]. Likewise, *HRAS* v.7 and v.8 are predicted to contain a novel initiation codon in exon 3 and, as a result, encode a new protein isoform that consists of 104 aa. The potential translation of these two alternative transcript variants would lead to the generation of a protein that displays a truncated N-terminal region, compared to the annotated HRAS isoform 2, and that lacks the effector lobe. Additionally, the newly identified *HRAS* v.9 and v.10 contain a smORF that is characterized by alternative initiation and termination codons, and the potential translation of these variants would lead to the generation of a small protein that may have regulatory roles [44]. Of note, *HRAS* v.11 and v.12 are predicted to encode a protein isoform that is identical to the annotated HRAS isoform 3 (110 aa), which is encoded by the annotated transcript variant 4 (Figure 4). The existence of three distinct transcript variants that encode the same protein isoform may indicate the tissue-specific expression of these mRNAs, as well as the differential regulation they undergo regarding their 5’ and/or 3’ untranslated regions.

Similar findings were observed from *NRAS*, as the obtained results led to the identification of nine novel *NRAS* transcript variants, seven of which are predicted to have ORFs and, thus, encode new protein isoforms. Particularly, it was found that the newly identified transcripts *NRAS* v.2, v.3, v.6, v.7 and v.8 contain previously undescribed smORFs and their potential translation would lead to the synthesis of NRAS micropeptides, containing less than 100 aa [44]. In specific, the protein encoded by *NRAS* v.3 is predicted to have a truncated effector lobe, but lacks the allosteric lobe, while the protein isoform that is encoded by the other four transcripts is expected to display no similarity to the annotated NRAS GTPase, since its amino-acid sequence is totally different. Additionally, *NRAS* v.4 is predicted to encode a novel protein isoform of 106 aa that retains the effector lobe of the G-domain but lacks the allosteric lobe. The generation of a protein that is characterized by an intact effector lobe without the site responsible for the stabilization of the nucleotide binding [14] would render the activation of the potential GTPase unlikely. Finally, the last coding transcript that was identified, *NRAS* v.5, is predicted to have the same ORF as the annotated *NRAS* v.1 and, thus, the potential translation of this particular novel variant would lead to the generation of the annotated NRAS GTPase (Figure 3).

In conclusion, our study highlights the wide spectrum of *RAS* mRNAs that are transcribed, providing an in-depth analysis of splicing events and exon/intron boundaries in a broad panel of cancerous and non-cancerous human cell lines. Although the current study provides a transcriptional profile regarding *KRAS*, *HRAS* and *NRAS*, further studies must be conducted to elucidate the biological function of all of the novel alternative transcript variants, as well as their putative protein isoforms.

## 4. Materials and Methods

### 4.1. Culture of Human Cell Lines

The implementation of the present work was carried out using an established panel of 40 human cell lines (Table S1). To investigate the expression of the putative novel RAS transcripts in a wide spectrum of human cancers, 37 cell lines that were cultured originated from 13 distinct human malignancies, whereas the other 3 are non-cancerous. All human cell lines were propagated based on the American Type Culture Collection (ATCC) guidelines.

### 4.2. Total RNA Extraction, Poly(A) Selection and First-Strand cDNA Synthesis

Total RNA isolation from every cultured cell line was carried out with the TRIzol Reagent (Ambion™, Thermo Fisher Scientific Inc., Waltham, MA, USA). The derived RNA samples were diluted in the RNA Storage Solution (Ambion™), whereas the assessment of their concentration and purity was performed spectrophotometrically at 260 and 280 nm, using a BioSpec-nano Micro-volume UV-Vis Spectrophotometer (Shimadju, Kyoto, Japan). Finally, oligo dT-based mRNA enrichment was performed using the NEBNext^®^ Poly(A) mRNA Magnetic Isolation Module (New England Biolabs Inc., Ipswich, MA, USA) and 5μg total RNA from each cell line.

The poly(A)-enriched RNA samples were used as templates in reverse transcription (RT) reactions for the generation of single-stranded cDNAs. For this purpose, an oligo-dT_20_ was used as an RT primer to anneal in the 3’ poly(A) tail of the mRNA transcripts. Briefly, each RT reaction was carried out in a Veriti 96-Well Fast Thermal Cycler (Applied Biosystems™) and in reaction volumes of 20 μL, which included 150 ng of mRNA, 1 μL of oligo-dT_20_ (10 μM), 1 μL dNTP Mix (10 mM each), 4 μL 5× First-Strand Buffer, 1 μL DTT (0.1 M), 1 μL RNaseOUT™ (Invitrogen™, Thermo Fisher Scientific Inc.) and 1 μL (200 U) of SuperScript™ III Reverse Transcriptase (Invitrogen™, Thermo Fisher Scientific Inc.), following the manufacturer’s protocol. The Glyceraldehyde 3-phosphate dehydrogenase (*GAPDH*) gene was used as housekeeping for the quality control of the produced cDNA samples.

Equimolar amounts of each cDNA sample were mixed for the creation of 14 distinct cDNA pools that corresponded to a specific human tissue or malignancy, as shown in Table S1. The derived cDNA pools were exploited as templates for the downstream PCR amplification step.

### 4.3. PCR Amplification of RAS mRNA Transcripts

Touchdown PCR-based assays were employed for the amplification of the mRNA transcript variants of *KRAS*, *NRAS* and *HRAS* genes in each cDNA pool sample with significantly increased sensitivity, specificity and PCR yield [49]. In specific, a forward (F) gene-specific primer was designed to target the first annotated exon and was used along with a reverse (R) gene-specific primer that was designed to anneal onto the last annotated exon of each targeted *RAS* gene (Table 1).

The touchdown PCR assays were performed in reaction volumes of 25 μL containing KAPA Taq Buffer A (Kapa Biosystems Inc., Wilmington, MA, USA), which included MgCl_2_ at a final concentration of 1.5 mM, 0.2 mM dNTPs, 0.4 μM of each primer, and 1 unit of KAPA Taq DNA Polymerase (Kapa Biosystems Inc.). In addition, the applied cycling protocol included an initial denaturation step at 95 °C for 3 min, followed by 30 cycles of 95 °C for 30 s, 65 °C (auto-ΔTa: −0.3 °C/cycle) for 30 s, 72 °C for 2 min, and a final extension step at 72 °C for 2 min. The amplicons from each cDNA pool sample were purified with the NucleoSpin^®^ Gel and PCR Clean-up kit (Macherey-Nagel GmbH & Co. KG, Duren, Germany).

### 4.4. Nanopore Sequencing

The purified PCR product derived from each cDNA pool was used for the construction of a barcoded library for nanopore sequencing. As a result, 14 barcoded libraries were constructed that corresponded to breast cancer, ovarian cancer, prostate cancer, colorectal cancer, urinary bladder cancer, hepatocellular carcinoma, lung cancer, cervical cancer, brain cancer, hematological malignancies, gastric cancer, endometrial cancer, renal cancer, and non-cancerous cell lines. Briefly, the NEBNext^®^ Ultra™ II End Repair/dA-Tailing Module (New England Biolabs, Inc) was employed for the end-repair process, the Agencourt AMPure XP beads magnetic beads (Beckman Coulter, Brea, CA, USA) were used for the nucleic-acid-purification steps, whereas the Quick T4 Ligase (New England Biolabs, Inc) enabled the adapter ligation. The derived barcoded libraries were quantified using the Qubit^®^ 2.0 Fluorometer and were mixed equimolarly. Nanopore sequencing was carried out on a MinION Mk1C sequencer (Oxford Nanopore Technologies Ltd., Oxford, UK), using a FLO-MIN106D flow cell with R9.4.1 chemistry and the Ligation Sequencing Kit (SQK-LSK109, ONT), following the manufacturer’s instructions.

### 4.5. Validation of Nanopore Findings with Next-Generation Sequencing

Reproducibility of the nanopore-sequencing findings was carried out using NGS based on a semi-conductor-sequencing technology. The Ion Xpress™ Plus Fragment Library Kit (Ion Torrent™, Thermo Fisher Scientific Inc.) was employed for the DNA-seq library construction, using 1 μg of purified PCR product mix as the input. Enzymatic fragmentation, adapter ligation, nick-repair and purification of the ligated DNA were implemented following the manufacturer’s protocol. Bead-based size selection of the constructed DNA-seq library was performed using the KAPA Pure Beads (Kapa Biosystems Inc.) in the recommended ratio of fragmented dsDNA:beads in order to enrich the library for 300–400 bp fragments. The size-selected library was quantified with the Ion Library TaqMan™ Quantitation Kit (Ion Torrent™) in an ABI 7500 Fast Real-Time PCR System (Applied Biosystems™). The sequencing template was created with emulsion PCR on an Ion OneTouch™ 2 System (Ion Torrent™), using the Ion PGM™ Hi-Q™ View OT2 kit (Ion Torrent™), whereas Ion OneTouch ES™ instrument (Ion Torrent™) was used for the template enrichment. Ultimately, a semi-conductor-sequencing methodology was carried out in an Ion 316™ Chip v2 using an Ion PGM™ System (Ion Torrent™) and the Ion PGM™ Hi-Q™ View Sequencing kit.

### 4.6. Post Processing and Bioinformatics Analysis

The primary analysis of the acquired nanopore-sequencing data, including base calling, demultiplexing, adapter trimming as well as quality assessment was performed with Guppy [50]. Nanopore-sequencing reads were separated into the “pass” and “fail” folder based on their quality scores, while only passed reads were used for the bioinformatics analysis. The obtained raw nanopore-sequencing data were mapped to the human reference genome (GRCh38) with Minimap2 aligner [51]. Mapped sequencing reads were visualized with IGV for the analysis of the existing alternative splicing events in the *RAS* target genes [52].

Besides mapping with Minimap2, the detection of alternative splicing events in the created FASTQ file was also implemented with our *in-house* developed algorithm “ASDT”, which was designed by members of our group as a generic splicing tool capable of identifying alternative splicing events and cryptic exons from high-throughput sequencing datasets [42]. Briefly, “ASDT” produces nucleotide k-mers for every possible splicing event that can occur between the annotated exons of a target gene. Then, it detects these nucleotide k-mers within the sequencing reads of the FASTQ file and extracts them into a separate output file. Furthermore, “ASDT” can detect novel exons, exon extensions or intron retentions by splitting intronic regions into a set of nucleotide k-mers and detecting them within the FASTQ file.

The raw number of the sequencing reads that cover the entire length of both the annotated and novel transcript variants was used to assess their abundance in human tissues. The derived count matrix was normalized using the median of ratios (MRN) method. The expression for each novel mRNA was calculated as the log2-transformed ratio of the difference between the normalized number of reads that corresponded to a specific transcript variant and the normalized number of all the annotated transcripts (log2FC).

In addition, a bioinformatics analysis of the acquired NGS dataset included the mapping of the experimental sequencing reads to the reference genome GRCh38 using HISAT2 aligner [53], the conversion of the created SAM file to BAM with samtools and, finally, the visualization of the aligned reads with IGV.

## Figures and Tables

**Table 1:** List of primers that were used for the specific amplification of the *RAS* mRNA transcripts with touchdown PCR. The melting temperature (T_m_) of each primer was calculated by Primer-BLAST designing tool.

Amplification of *RAS* Transcripts with Touchdown PCR	Primers			
	Direction	Sequence (5′→3′)	Length (nt)	Tm (°C)
*KRAS*	Forward	GCCATTTCGGACTGGGAGC	19	61.12
	Reverse	CTCGAACTAATGTATAGAAGGCATCATC	28	59.76
*NRAS*	Forward	GCTGTGGTCCTAAATCTGTCCA	22	60.03
	Reverse	CTGTGAGACTGAAGACAGCAAC	22	59.20
*HRAS*	Forward	GCAGTCGCGCCTGTGAAC	18	62.08
	Reverse	GCACCTCCATGTCCTGAGCTT	21	62.40

## Data Availability

The described novel nucleic acid sequences of the present work, which correspond to the new *KRAS*, *NRAS* and *HRAS* cDNA sequences (*KRAS* v.5 – v.20, *NRAS* v.2 – v.10 and *HRAS* v.5 – v.18), have been submitted and deposited to the GenBank^®^ Data Library, under the accession numbers MZ068300–MZ068338, accordingly.

## Supplementary Materials

The following are available online at https://www.mdpi.com/article/10.3390/ijms222413298/s1.
