# Supplementary material for: Targeted Long-Read Sequencing Decodes the Transcriptional Atlas of the Founding RAS Gene Family Members
Source: Int J Mol Sci. 2021 Dec 10;22(24):13298. doi: 10.3390/ijms222413298 (PMC8709048; doi:10.3390/ijms222413298)
Supplement: Supplementary file 1 [file ijms-22-13298-s001.zip › Supplementary Data.pdf]

Nanopore sequencing reads confirming novel *KRAS mRNA transcripts*

***KRAS* v.5** | Exon 1 (179 bp) / Exon 2 / Exon 4 / Exon 6 | @a257cbe2-2399-4af5-bec3-ff0846f33c87

GCCATTTCCGGACTGGGAGCAGGCGCGGCGCAGGCACTGAAGGCGGCGGCGGGGCCAGAGGCTCCAGCGGCTCCAGGTGCGGGAGAGAGGCCTGCTGAAAATGACTGAATATAAACTTGTGGTAGTTGGAGCTGGTGGCGTAGGCAAGAGTGCCTTGACG
ATACCAGCTAATTCAGAATCATTTGTGGACGAAATATGATCCAACAATAGAGAGAACAAATTAAAAGAGTTAAGGACTCTGAAGATGTACCTATGGTCTAATTAGGAAATAAATGTGATTTGCCTTCTAGAACAGTAGACACAAAACAGGCTCAGGACTT
AGCAAGAAGTATGGAATTCCTTTTATTGAAACATCAGCAAAAGACAAGACAGGGTGTTGATGATGCAGCCTATACA

***KRAS* v.6** | Exon 1 (166 bp) / Exon 2 / Exon 4 / Exon 6 | @f1e3643f-775d-45e6-bfb1-55626332e2ae

ATTTCGGACTGGGAGCGAGCGCGGCGCAGGCACTGAAGAAGGCGGCGGCGGGGCCAAGGGGCTCAGCGGCTCCCAGGCCTGCTGAAAATGACTGAATAAACGGTAGTTGGAGCTGGTGGCGTAGGCAAGAGTGCCTTGTTGATACAGCTAATTCGAGTCA
TTTTGTGGTTGAATATGATCCAACAATAGAGAGAACAAATTAAAGAGACAAGGACTCTGAAGTGTACCTATAGTCCTAGTGATAAATGTGATTTGCCTTCTAGAACAGTAGACACAAAAACAGGCTCAGGGACTTAGCAAGAAGTTATCGATTCCTTTTA
TTGAAACATCAGCAAAGACAAGACAGGGTGTTGATGATGCCTTCTATATACAGTAGTTCGAGAGCAATACGTAACCCG

***KRAS* v.7** | Exon 1 (179 bp) / Exon 2 / Exon 4 / Exon 5 / Exon 6 | @8739cda9-2052-4834-a963-fcfdf6ecab9b

GCGGGGCCGACTCCAGCGGCTCCCAGGTGCGGGAGAGAGGCCTGCTGAAATGACTGAATAAACTTGTAGTAGTTGGAGCTGGTGGCGTAGGCAAGAGTGCCTTGACGATACAGCTAATTCAGAATCATTTTGTGGACGAATATGATCCAACAATAGAGAG
AACAAATTAAGAGTTAAGGATACGAAGATGTACCTATATAGTCTATTAGGAAATAAATGTGATTTGCCTTCTAGAACAGTAGACACAAAACAGGCTCAGGACTTGAAGTTATGAATTCCTTTATTTGAAACATCAGCAAAGACAAGACAGAGAGTGGAGG
ATGCTTTTTTATACATTGGTGAGAGAAGTCCGACAATACAGATTGAAAAAATCAGCAAGAAGAAAAGACTCTGGCTGTGTGAAAATTAAAAAATGCATTATAATGTAATCTGGGTGTTGATGATGCCTTCTACATTGGATTCGAGAGCAATACGTGGCA

***KRAS* v.8** | Exon 1 (166 bp) / Exon 2 / Exon 4 / Exon 5 / Exon 6 | @33743cba-c72e-430e-bfea-0dc0a5a8f368

CAGGCACTGAAGGCAGCGGCGGGGCCAGAGCTCCAGCGGCTCCCAGGCCTGCTGAAAATGACTGAATATAAACTTGTGGTAGTTGAGCTGGTGGCGTAGGCAAGAGTGCCTTGACGATACAGCTAATTCAGAATCATTTTGTGGACAGGTATGATCCAAC
AATAGAGAGAACAAATTAAAGAGTTAAGGACTCTGAAGATGTACCTATGGTCCTAGTAGGAATAAATGTTGATTTGCCTTCTAGAACAGTAGACACAAAACAGGCTCAGGACTTAGCAAGAAGTTATGGAATTCCTTTTATTGAAACATCAGCAAAGACA
AGACAGAGAGTGGAGGATGCTTTTTATACATTGGTAGAGAGAGATCCGACAATACAGATTGAAAAAAATCAGCAAAGAAGAAAAAGACTCCTGGCTGTGTGAGGAATTAAAATGCATTATAATGTAATCTGGGTGTTGATGATGCCTTCTATACACATTA

***KRAS* v.9** | Exon 1 (166 bp) / Exon 2 / Exon 5 / Exon 6 | @ce1f8047-c967-47c8-9ef5-1985daed4e4c

GTTGTACTTCGTTCAGTTACGTATTGCACCGACGTACAGGGAGCGAGCGCGGCGCCCAGGCACTGAAGGCGGCAGCGGGAATGACTCAGCGGCTCCCAGGCCTGCTGAAAATGACTGAATATAAAGCTGGCGGTAGTTGGAGCTGGTGGCGTAGGCAGAG
TGCCTTGACGATACAGCTAATTCAGGGCATCATTTTGTGGACGAATATGATCCAACAATAGAGAGAGTGGAGGATGCTTTTATACATTGGTGAGAGATCCGACAATACAGATTGAAAAATCAGCAAGAAGAAAAAGACTCCCTGGCTGTGTGAAAATTA
AAAATGCATTATAATGTAATCTGGGTCATTGATGCCTTCCTATACATTAGTTCGAGAGCAATACACGTAACTA

***KRAS* v.10** | Exon 1 (179 bp) / Exon 2 / Exon 6 | @d8888cff-5587-4363-8897-316b12ee2788

CTTCGTTCAGTTACGTATTGCTGCCATTTCGGACTGGGGCAGGCGCGGCGCAGGCACTGAAGGCGGCAGCGGGATTGCAGAGCTCAACGGCTCCCAGGTTGCGGGAGAGAGGCCTGCTGAAATGACTGGTATAAACTTGTGGTAGTTGGGCTGGTGGCGT
AGGCAAGAGTGCCTTGACGATACAGCTAATTCAGAATCATTTTGTGGACGAATATGATCCAACAATAGAGGGTGTTGATGATGCCTTCTATACATTAGTTCAGGAAACAGCCAACA

***KRAS* v.11** | Exon 1 (166 bp) / Exon 2 / Exon 6 | @e794dd57-94cd-4974-85ea-f56f5f00bb13

GGTATGCTTCGTTCAGTTACGTATTGCTGCCATTTCGGACTGGGAGCGAGCGCGGCATGAGCACTGAAGGCGGCAGCGAGACCAGGCTCAGCGGCTCCCAGGCCTGCTGAAAATGACTGAATATAAACTTATGTTAGTTGGGCTGGTGACGTAGGCAAGG
TGCCTTGACGATACAGCTAATTCAGAATCATTTTGTGGACGAATATGATCCAACAATAGAGGGTGTTGATGATGCCTTCTATACGTGTTAGTTCGAGAAGCAATACGTAACCTT

***KRAS* v.12** | Exon 1 (179 bp) / Exon 3 / Exon 4 / Exon 6 | @b08afd94-b236-451e-9bc4-f1e2d7e263fa

GCTGCCATTTCGGACTGGGGGCAGGCGCGGCGCAGGCACTGAAGGCGGCGGCGGGACTGAGGCTCAGCGGCTCCCAGGTGCGGGAGAGAGGATTCCTACAGGAAGCAAGTAGTAATTGATGGAGAAACCTGTCTCTTGGATATTCTTGACACAGCAGGTC
AGAGAGTACAGTGCAATGAGGGACCAGTACATGAGGGACTGGGGCTTTCTTTGTGTATTTGCCATAAATAATACTAAATCATTTGAAGATATTCACCATTATAGAGAACAAATTAAAAGAGTTAAGGACTCTGAAGATGTACCTATGGTCCTAGTAGGAA
ATAAATGTGATTTGCCTTCTAGAACAGTAGACACAAAACAGGCTCAGGACTTAGCAAGAAGAAGTTATGGAATTCCTTTTATTGAAACATCAGCAAAGACAAGACAGGGTGTTGATGATGCCTTCTATACATTAGTTCGAGAGAGCAATACGTAACCAA

***KRAS* v.13** | Exon 1 (166 bp) / Exon 3 / Exon 4 / Exon 6 | @2aeee591-44fe-4222-a3b7-e8baad295d9d

CCAGTTACGTATTGCTGCCATTTCGGACTGGGAGCAGCGCAGCGCAGGCACTGAAGGCGGCAGCGGGGCCAGAGGCTCAGCGGCTCCCAGGATTCCTACAGGAAGCAAGTAGTAATTGATGGAAGAAACCTGTCTCTTGAGATATTCTCGACACAGCAGG
TCAAAGAGGAGTACAGTGCAATGAGGGACCAGTACACATGAGGACTGGGAGGGCTTCTTTGTGTATTTGCCATAAATAATACTAAATCATTTGAAGATATTCACCATTATAGAGAACAAATTAAAAGGTTAAGGACTCTGAGATGTACCCTATGGTCTAG
TAGGAAATAAATGTGATTTGCCTTCTAGAACAGTAGACACAAAACAGGCTCAGGACTTAGCAAGAAGTTATGGAATTCCTTTTATTGAAGAACATCAGCAAAGACAAGACAGGGTGTTGATGATGCCTTCTATACATTAGTTCGAGAGCAATACGTAGC

***KRAS* v.14** | Exon 1 (179 bp) / Exon 3 / Exon 4 / Exon 5 / Exon 6 | @71eb8340-8ada-48f2-b446-54e0c76c1958

ACCGAAGGCGGCGGCGGGCCAGAGGCTCAGCGGCCCCCAGGTGCGGGAGAGAGGATTCCTACAGGAAGCAAGTAGTAATTGATGGAGAAACCTGTCTTCTGGATATTCTCGACACAGCAGGTCAAGAGGAGTACAGTGTAATGAGGGACCAAAGCATGAG
GACTGGGAGGGCTTTCTTTGTGTATTTGCCATAAATAATACTAAATCATTTGAAGATATTCACCATTATAGAGAACAAATTAAAAGAGTTAAGGACTCTGGAAGATGTGTACCATGGTCCTAGTAGGAAATAAATGTGATTTGCCTCTAGAACAGTAGAC
ACAAAACAGGCTCAGGACTTAGCAAGAAGTTATGGAATTCCTTTATTGAAACATCAGCAAAGACAAGACAGAGAGTGGAGGATGCTTTTTATACATTGGTGAGAGAGATCCGACAACAGATTGAAAAATCAGCAAAGAAGAAAAGACTCCTGGCTGTGTG
AAAATTAAAAATGCATTATAATGTAATCTGGGTGTTGATGATGCCTTCTATACATAGTTCGAGAGCAATAC

***KRAS* v.15** | Exon 1 (166 bp) / Exon 3 / Exon 4 / Exon 5 / Exon 6 | @bc424705-b50b-44a0-8eb2-9fed9643ed65

ACGCGCGCAGGCACTGAAGGCGGCGGCGGGCCAGAGGCTCAGCGGCTCCCAGGATTCCTACAGAAGCAAGTAGTAATTGATGGAGAAACCCTGTCTTTGGATATTCTCGACACAGCAGGTCAAGAGGAGTACAGTGCAATGAGGGACCAGTATGAGGATC
AGGAGGGCTTTTCTTTGTGTATTTGCCATAAATAATACTAAATCATTTGAAGATATTCACCATTATAGAGAACAAATTAAAAGAGTTAAGGACTCTGAAGATGTACCTATGGTCCTAGTAGGAAATAAAATGTGATTTGCCTTCTAGAACAGTAGACACA
AACAGGCTCAGGACTTAGCAAGAAGTTATGGAATTCCTTTATTGAACATCAGCAAAGACAAGACAGAGAGTGGAGGATGCTTTTTATACATTGGTGAGAGAGATCCGACAATACAGATTGAAAAAAAAAATCAGCAAAGAAGAAAGACTCTGGCTGTGTG
TGAAAATTAAAAATGCATTATAATGTAATCTGGGTGTTGATGATGCCTTCTATACATTAACGAGAGCAATAC

***KRAS* v.16** | Exon 1 (179 bp) / Exon 4 / Exon 6 | @f89587bd-2d2f-4ea5-9362-01f078d26137

CGGACTGGGAGCAGGCGCGGCTGAGCACTGAAGGCGGCGGCGGCAGGACCAGAGGCTCAGCGGCTCCCAGGTGCGGGAGAGAGAGAACAAATTAAAGAGTTAAGGACTCTGAAGATGTACCACATGGTCTAGTAGGAAATAAATCGTGGGTTTGCCTTCT
AGAACAGTAGACACAAAACAGGCTCAGGACTTAGCAAGAGTTATGGAATTCCTTTTATTGAAACATCAGCAAAAGACAAGACAGGGTGTTGATGATGCCTTCTATACATTAGTTCGAGAATAATACGTAACCT

***KRAS* v.17** | Exon 1 (166 bp) / Exon 4 / Exon 6 | @6d4c1935-08d2-46f3-8e90-dc8766e81288

GGTGTACTTCGTTCAGTTACGTATTGCTGCCGACGGACTGGGAGCCACTGAAGGCGGCAGCGGGGCCCAGAGCTCCAGCGGCTCCCAGAGAACAAATTAAAAGAGTTAAGGACTCTGAAGATGTTACCTATAGTCCCTGATAGGAAATAAATGTGATTTG
CCTTCTAGAACAGTAGACACAGAACAGGCTCAGGACTTAGCAAAGTTATGGAATTCCTTTTATTGAAACATCAGCAAAGACAAGACAGGGTGTTGATGATGCCTTCTATACATTAGTTCGAGAGCAATACGTAA

***KRAS* v.18** | Exon 1 (179 bp) / Exon 4 / Exon 5 / Exon 6 | @87e6ba6f-6c60-4909-9cff-449fbdf34f3e

ATCGGGAGCGAGTGCGGCGCAGCAAGCACTGAAGGCGGCGGCGGGGCCAGAGGCTCAGCGGCCTCAGGTGCGGGAGAGAGAGAACAAATTAAAAGAGTTAAGGACTCTGAAGATGTACCTATGGTCCTAGTAGGAAATAAATGATTTGCCTCTAGAACAG
TAGACACAAAACAGGCTCACAGGACTTAGCAAGAAGTTATGGAATCTCTTTATTGAAACATCAGCAAAGACAAGACAGAGAGTGGAGGATGCTTTTATACATTGGGTGAGAGATCCGACAATACAGATTGAAAAAATCAGCAAAGAAGAAAAGACTCC
TGGCTGTGTGAAAATTAAAAAATGCATTATAATGTAATCTGGGTGTTGATGATGCCTTCTATACATTAGTTCGAGAGCAATATGTAACTGAACGAAGTACAAC

***KRAS* v.19** | Exon 1 (179 bp) / Exon 5 / Exon 6 | @5fc070c3-be7b-43a3-a4e0-55e64aea8555

TGCCATTTTCGGACTGGGAGCGAGCGCGGCGCAGGCACTGAAGGCGGCAGCGGGGCCAGAGAGGCTCAGCGGCTCCCAGGTGCGGGAGAGAGAGAGTGGAGGATGCTTTTTATACATTGGTGAGAGATCCGACAATACAGATTGAAAAAAAAATCAGCAA
GAAGAAAAAGACTCCTGGCTGTGTGAAAATTAAAAAATGCATTATAATGTAATCTGGGTGTTGATGATGCCTTCTATACATTAGTTCGAGAAGCAATACGTAACCCCT

***KRAS* v.20** | Exon 1 (179 bp) / Exon 6 | @34d23ef7-2d03-44da-8148-3bc342337722

GCTGCCATTTCGGACTGGGAGCAGGCGCGGCGCAGGCATGAGGCGGCGGGATAGAGGCTCAGCGGCTCCCCGGGTGCGGGAGAGAGGGTGTTGATGATGCCTTCTATGCATTAGTTCGAGAAGCAATACGTAACTTG

Nanopore sequencing reads confirming novel *NRAS mRNA transcripts*

NRAS v.2 | Exon 1 / Exon 2 / Exon 4 / Exon 5 / Exon 6 / Exon 7 | @4818787d-d10d-4553-ab0f-2f14c8e9e7cb

GTTATTACTTACCGTTCAGTTACGTATTGCTGCTGTGGTCCTAATCCTGTCCAAAGCAGAGAGGCAGTGGAGCTTGAGGTTCTTGCTGGTGTGAAATGACTGGAGTACAAACTGAAGGTGTTGATAGTTAGGGCGGGTGGTGTTGGGAAAGCGCACTGAC
AATCCAGCTAATCAAGATTTCCACTTTGTAGATGAATATGATCCACCATAGAGGGAGCAGATTAAGCGAGTAAAGAACTCGGATGATGTACCTATGGTGCTGAGTGGGAAACAAGTGTGATTTGCCAACAAGGACGGATTGATACAAAACAAGCCCACGA
ACTGGCCAGAGTTACGGGATTCCATTCATTGAAACCTCAGCCCAAGACCAGACAGGGTGTTGAAGATGCTTTTACACACTGGTAAGAGAAATACGCCAGTACCAGATGAAAAAAACTCAACAGCAGTGATGATAGAGTAGGCTCAGGTTGTATGGGATTG
CCATGTGTGGTGATGTAACAAGATACTTTTAAAGTTTTGTCAGAAAAGAGCCACTTTCAAGCTGCACTGACACCCTGGTCCTGACTTCCCTGGAGAATATTCCTGTTGCTGTCTTCAGTCTCACAGGTAATACGTAACTA

NRAS v.3 | Exon 1 / Exon 2 / Exon 5 / Exon 6 / Exon 7 | @b3b02955-cf9d-4127-a42f-12767d51b28b

GTTATTACTTCGTTCAGTTACGTATTATTCACTGCATTGCTGTGGTCCTAAATCTGTGTCCAAAGCAAAGGCAGTGGAGCTTGAGGTTCTTGCTGGTGTGAAATGACTGAGTACAAACTGGTGGTGGTTAAGACGGGTGGTGTTGGGAAAGCGCACTGAC
AATCCAGCTAATCCAGAACCACTTTGTAGATGAATATGAATCCACCACCATAGAGGGTGTTGAAGATCACTTTTTTACACTGGTAGAGAAATACGCCAGTACCGAATGAAAAAAAAACTCAACAGCAGTGATGATGGGACTCAGGGTTGTATGGGATTGC
CATGTGTGGTGATGTAACAAGATACTTTTAAAGTTTTGTCAAGAAAAGAGCCACTTTCAAGCTGCACTGACACCTGGTCCTGACTTCCCTGGAGGAGAGAATATTCCTGTTGCTGTCT

NRAS v.4 | Exon 1 / Exon 2 / Exon 3 / Exon 5 / Exon 6 / Exon 7 | @0b40509c-9701-48f1-b797-046823b3bcb4

TATTACTTCGTTCAGTTACGTATTGCTGCCACATGGTCTAAATCTGTCCAAACAGAGGCAGTGTGGAGCTTGAGGTTCTTGCTGGTGTGAAATATTTGAGCAAATAATTGATGAGGATTTGGAGCAGGTGGTGTTGGGAAAGCGCACTGACAGTCCAGCT
AATCAGAACGCCACTTTGTAGATGAATATGATCCCACCATAGAGGATTCTTACAGAGAAAACAAGTGGTTATAGATGGTGGGAAACCTGTTTGTTGGACATACTGGATACAGCTGGACAAGAAGAGTACAGTGCCATGAGAGACCAATACATGAGGACAG
GCGAGAAACTTCCTCTGTGTATTGCCATCAATAATAACTTAAGTCATTTGCGGATATTAACCTCTACAGGGTGTTGAAGATGCTTTTACACACACTGGTAAAGAGAGAAATACGCCAGTACTGAATGAAAAAACTCAACAGCAGTGATGATGATGGGACT
CAGGGTTGTATGGGATTGCCATGTGTGGTGATGTAACAAGATACTTTTAAAGTTTTGTCAGAAAGAGCCACTTTCAAGCTGCACTGACACCCTGGTCCTGACTTCCCTGAATATTCCTGTTGCTGTCTTCAGTCTCACAGATCAATACGTAACCT

NRAS v.5 | Exon 1 / Exon 2 / Exon 3 / Exon 4 / Exon 5 / Exon 7 | @490e0bbf-14f0-464e-8ebe-89467dfc0618

TCAGTGTACTTCGTTCAGTTACGTATTGCTGCTGTGGTCTAAATCTGTCCAAAGCAGAGGCAGTGGAGCTTGAGGTTCTTGCTGGTGTGAAGTGACTGAGTACAAACTGGGTGGTGAGGTTGGAGCAGGTGGTGTTGGGAAAAGCGCACTGACAGTCAGC
TAATCCAGAACCACTTTGTAGATGAATATGATCCCACCATAGAGGATTCTTACAGAAAACAAGTGGTTATAGATGGGTGAAACCTGTTTGTTGGACATACTGGATACAGCTGGACAAGAAGAGTACAGTGCCATGAGAGACCAATACATGAGGACAGGCG
AAGGCTTCCTCTGTGTATTGCCATCAATAATAGCAAGTCATTTTGCGGATATTAACCTCTACAGGGAGCAGATTAAGCGAAAGGTAAGAGACTGGATGATGATGTACCTATGGTGCTAGTGGGAAGCAAGTGTGATTTGCCAACAAGGACAGTTGATACA
AAACAAGCCCACGAACTGGCCAAGAGTTACAGGATTCCATTCATTGAAACCTCAGCCAAGACCAGACAGGGTGTTGAAGATACTTTTACACACTGGTAAGAGAAATATATATTACCGAATGAAAAAACTCAACAGCAGTGATGATGGGACTCAGGGTTGT
ATGGGATTGCCATGTGTGGTGATGTAACAAGCTGCACTGACACCCTGGTCTGGCTTCCCTGGAGGAGAAGTATTCCTGTTGCTGTCTTCAGTCTCACAGAGTATATTACGTAA

NRAS v.6 | Exon 1 / Exon 3 / Exon 4 / Exon 5 / Exon 6 / Exon 7 | @716cf80d-6aa3-4997-8218-c499d5109a9d

GTGTACTTCGTTCAGTTACGTATTGCTGCTGTGGTCCTAAATCTGTCCAAAGCAGAGGCGTGGAGCTTGAGGATTCTTACAGAAAGCAGAGTGGTTATAGATGGTAGAAACCTGTTTGTTGGACATACTGGATACAGCTGGACAAGAGAAGAGTACAGTG
CCATGAGAGACCAATACATGAGGACAGGCGAGAAGGCTTTGTGTTGCCATCAATAATAACTTAAAGTCATTTGCGGATACCAACCTCTACAGGGAGCAGATTAAGCGAGTAAAAGACTCCGGATAGATGTACCTATGGTGCTAGTGGGAAACAAGTGTGA
TTTGCCAACAAGGACAGTTGATACAAAACAAGCCCACGAACTGGCCAAGAGTTACGGGATTCCATTCATTACAAGAGCCTCAGCCAAGACCAGACAGGGTGTTGAAGATGCTTTTTTACACACTGGTAAAGAGAAATACGCCAGTACCGAATGAAAAAAC
TCAACAGCAGTGATGATGGGACTCAGGGTTGTATGGGATTGCCATGTGTGTGGTGATGTAACAAGATACTTTTAAAGTTTTGTCAGAAAAAGGCCACTTTCAAGCTGCACTGACACCCTGGTCCTGACTTCCCTGGAGGAGAAGTATTTCCTGTTGCTGT

NRAS v.7 | Exon 1 / Exon 4 / Exon 5 / Exon 6 / Exon 7 | @6b8cac39-0713-4acb-bef9-5cb90efefd65

TTGTTGTGGTCCCAAATCTGTCCAAAGCAGAGGCGGTGGAGCTTGAGGGAGCAGATTAAGCGGTAAAGACTGGATGATGTACCTATGGTGCTAGTGTGTCAAGTGTGATTTGGCCAACAAGGACAGTTGATACAAACAAGCCTCCACGAACTGGCCAAGA
GTTACGGATTCCATTCATTGAAACCTCAGCCAAGACCAGACAGGGTGTTGAAGATGCTTTTTACACACTGGTAAGAGAAATACGTCGAAAAAACTCAACAGCAGTGATGATGGACCAGGGTTGTATGGGATTGCCATGTGTGGTGATGTAACAAGATACT
TTTAAAGTTTTGTCAGAAAAGAGCCACTTTCAAGCTGCACTGACACCCTGGTCCTGACTTCCTGAGGAGAAGTATTCTGTTGCTGTCTTCAGTCTCACAGAGCAATACGTAACTGAACGAAGTACACGC

NRAS v.8 | Exon 1 / Exon 4 / Exon 5 / Exon 7 | @f3d42699-86ea-49ae-a4e7-49dec26fcc10

TAATCCAAAGCAGAGGCAGTGGAGCTTGAGGGAGCAGATTAAGCGAGTAAAGACTCGGATGATGTACCTATGGTGTTAGTGGGAAACAAGTGTGATTTGCCAACAAGGACAGTTGATACAAAACAAGTCTAATGAACTGGCCAAGAGTTACGGGATTCAT
TCATTGAAACCTCAGCCAAGACCAGACAGGGTGTTGAAGATGCTTTTACACACTGGTAAGAGAAATACGCCAGTACCGAATGAAAAAACTCAACAGCAGTGATGATGGGACTCAGGTTGTATGGGATTGCCATGTGTGGTGATGTAACAAGCTGCACTGA
CACCCTGGTCCTGACTTCCTGGAGGAGAAGTATTCCTGTTGCTGTCTTCAGTCTCACAGAGCAATACGTAACTGAACGAAGTACAAC

NRAS v.9 | Exon 1 / Exon 5 / Exon 6 / Exon 7 | @84f0d693-cef4-41f8-a08a-0e8d9c8c016f

GTATACTTCGTTCAGTTACATATTGCTGCTGTGGTCCCTGAAATCTGTCTCAAAGCGAGGCAGTGGAGCTTGAGGGTGTTGAAGATGCTTTTTACACACTGGTAAAATACGCCAGTACCGAATGAAAAAACTCAACAGCGTGATGATGGGACTCTCAGGA
GTTGTATGGGATTGCCATGTGTGTGGTGATGTAACAAGATACTTTTAAAGTTTTGTCAGAAAGAGCCACTTTCAAGCTGCACTGACACCTGGTCTGACTTCCCTGGAGGAAGTATTCCTGTTGAGTTTTTCATTCGACTACTAACGCCTTCCCTAACACA

NRAS v.10 | Exon 1 / Exon 5 / Exon 7 | @fe0d1a78-3525-475f-a467-be61735ec46d

TATTTTTTTTTTTCGATATTGCTGCTGTGGTCCTAAATCTGTCCAAAGCAGGCAGTGGAGCTTGAGGGTGTTGAAGATGCTTTTTACACACTGGTAAGAGAAATACGCCAGTACCGAATGAAAAACTCAACAGCAGTGATGATGGGACTCAGGTTGTATG
GATTGCCATGTGTGGTGATGTAACAAGCTGCACTGACACCCTGGTCCTGACTTCTGGAGGAGAAGTATTCCTGTTGCTGTCTTCAGTCTCACAGAGCAATACGTAACTGAACGAAGTACAAC

Nanopore sequencing reads confirming novel *HRAS mRNA transcripts*

***HRAS* v.5** | Exon 1 / Exon 3 / Exon 4 / Exon 6 / Exon 7 | @f73429e7-260c-433a-8ae0-0820eb4d56d7

GCAGTCGCGCCTGTGAACGGATTCCTACCGGAAGCGGTGGTCATTGATGGGGAGACGTGCCTGTTGGACATCCTGGATACCGCCGGCCAGGAGGAGTACAGCGCCATGCAGGACCAGTACATGCGCACCGGGGAGGGCTTCCTGTGTGTGTTTGCCATCA
ACAACACACCAAGTCTTTTGAGGACATCCACCAGTACAGGGAGCAGATCAAACAGGTGAAGGACTCGGATGACGTGCCCATGGTGCTGGTGGGGAACAAATTGTGACCTGGCTGCGCACTGTGGAATCTCGGCAGGCTCAGGACCTCGCCGAAGCTACGG
CATCCTACATCGAGACCTCGGCCAGAGACCCGGCAGGGAGTGGAGGATGCCTTCTACACGTTGGTGCGTGAGATCCCGGCAGCACAAGCTGCGGAAGCTGAACCCTCTGATGAGAGTGGCCCCGGCTGCACCCAGGCTGCAAGAAGTGTGGCTCTCCTGA
CGCAGCACAAGCTCAGGACATGGAGGTGC

***HRAS* v.6** | Exon 1 / Exon 3 / Exon 4 / Exon 6 (514nt) | @36113edd-b014-40e3-b768-5fc99497e015

GCAGTCGCGCCTGTGAACGGATTCCTACCGGAAGCAGGTGGTCATTGATGGGGAGACGTGCCTGTTGACATCCTGGATACCGCCGGCCAGGAGGAGTACAGCCATGCGGGACCAGTACATGCGCACCGGGGAGGGCTTCCTGTGTGTGTTTGCCATCAAC
AACACCAAGTCTTTGAGGACATCCACCAGTACAGGGAGCAGATCAAACGGGTGAAGGACTCGGATGACGTGCCCATGGTGCTGGTGGGGAACAGTGACCTGGCTGCACGCACTGTGGAATCTCGGCAGGCTCAGGACCTCGCCTGAAGCCACGGCATCCT
ACATCGAGACCCGGCCAAGACCCGGCAGGGAGTGGAGGATGCCTCTACACGTTGGTGCGTGAGATTCGGCAGCACAAGCTGCGGAAGCTGAACCTCCTTGATGAGAGTGGCCCCACTGCATGTGAGCTGCAGCGTGTGCTCTCCTGACGCAGGTGAGGGG
GACTCCCAGGGCGGCCGCCACGCCCACCGGATGACCCTGGCTCCCGCCCCTGCCGGTCTCCTGGCCTGCGGTCAGCAGCCTCCCTTGTGCCCCGCCCAGCAATGCAAGCTCAGGACATGGAGGTGC

***HRAS* v.7** | Exon 1 / Exon 3 / Exon 4 / Exon 5 / Exon 6 / Exon 7 | @d349de3a-d747-4cbd-9aa2-89427fdb21d5

GCAGTCGCGCCTGTGAACGGATTCCTACCGGAAGCGGTGGTCATTGATGGGGAGACGTGCCTGTTGGACATCCTGGATGATACCGCCGGCCAGGAGAATTACAGCGCCATGCAGGACCAGTACATACGCACCAGGGAGGAACTTCCCTGTGTGTGTTTGC
CATCAGCAACGCCAGTCTTTTGAGGACATCCACCAGTACAGGGAGCAGATCAAACGGGTGAAGGACTGGATGACGTGCCCATGGTGCTGGTGGGAACAAATGTGACCTGGCTGCACCGCACTGTGGAATCTCGGCAGGCTCAGGACCTCGCCAGAAGCTA
CATCCCCTACATCGAGACCTCGTGACCCGGCAGGGCAGCCGCTCCTGTTCACCCTAGGACCCCCGGGACCCGGTGCCCCTCGCGCTGGAGTGGAGGATACCTTCTACACGTTGGTGCGTGAAGATCCGGCAGCACAAGCTGCGGAAGCTGAACCCTCTGA
TGAGTGGCCCCATGTGAGCTGCAAGTGTGTGCTCTCCTGACGCAGCACAAGCTCAGGACATGGAGGTGC

***HRAS* v.8** | Exon 1 / Exon 3 / Exon 4 / Exon 5 / Exon 6 (514nt) | @49895350-4d45-4a10-904a-32d719d51907

GCAGTCGCGCCTGTGAACGGATTCCTACCGGAAGCAGGTGGTCATTGATGGAGACGTGCCTGTTGGACATCCTGGATACCGCCGGTAGCCAGGAGGAGTACAGCGCCATGCGGGACCAGTACATGCGCACCGGGGAGGGAGCTTCCTGTGTGTTTGCCAT
CAACAACACCAAGTCTTTTGAGACATCCACCAGTACAGGGAGCAGATCAAACGGGTGAAGGACTCGGATGACGTGCCCATAGTGCTGGTGAGAACAAAGTGTGACCTGGCTGCACGCACTGTGGAATCTCGGCAGGCTCAGGACCTCACCGCCCGAAGCT
ACAGCATCCCCATCGAGACCTCGGCCAAGACCCGGCAGGGCAGCCGCTCTGGCTCTAGCTCCAGCTCCGGGACCCTCTGGGACCCCGGGACCCACTGTGACCCATGGCCCCTCGCGCTGGAGTGGAGGATGCACTTCTACACGTTGGTGCGTGAGATCCG
GCAGCACAAGCTGCGGAAGCTGAACCCTCTGATGAGAGTGGCTGGCTGCATGAACAGAGTGTGTGCTCTCCTGACGCAGGTGAGGACTCCCAGGGCCGCCACGCCCACCGGATGACCCCGGCTCCCCGCCCCTGCCGGTCTCCTGGCCTGCGGTCAGCCT
CCCTTGTGCCCCGCCCAGCACAAGCTCAGGACATGGAGGTGC

***HRAS* v.9** | Exon 1 / Exon 4 / Exon 6 / Exon 7 | @58e06c3d-9190-4221-915a-ad91acddb2dd

GCAGTCGCGCCTGTGAACGGGAGCAGATCAAACGGGGTGAAGGACTCGGATGACGTGCCCATGGTGCTGGTGGAAATGCGTGAGTTCCGGCTTACCGCACTGTGGAATCTCGGCAGGCTCAGGACTCGCTCGAAGCTACGGCATCCCCTACATCGAGACC
TCGGCCAAGACCCGGCAGGGAGTGGAGGATGCCTTCTACACGTTGGTGCGGAGATCCGGCAGCAATAAGCTGCGAAGCTGAACCTCCTGGATGAGAGTGGCCCTGCTGCATGAGCTGCAAGTGTGTAAGCCTCTCCTGACGCAGCACAAGCTCAGGACAT
GGAGGTGC

***HRAS* v.10** | Exon 1 / Exon 4 / Exon 6 (514nt) | @7aa1fc14-8bfb-4d12-beb3-7c58db012db4

GCAGTCGCGCCTGTGAACGGGAGCAGATCAAACGGGTGAAGGACTCGGGATGACGTGCCCATGGTGCTGCGGGGAACAGTGTGACCTGGCTGCACGCACTGGGGAATCTCGGCAGGCTCAGGACTGCCCGAAGCTACGGCATCCCCTACATCGAGACCTC
GGCCAAGACCCGGCAGGGAGTGGAGGATGCCTCTGCCATCGTTGGTGCGGGAGGATCCGGCAGCAATAAGCTGCGGGAAGCCGGAACCCCTCCCTGATGAGAGTGGCTCCCGGCTGCATGAGCCGGTGGGGTGTGTGCCTCCTGACGCAGGTGAGGGGAC
TCCCAGGCGGCTGCCACGCCCACCGGATGACCCGGCTCCCCGCCCTGCCGGTCTCCTGGCCTGCGGTCAGCAGCCCCTTGTGCCCCGCCCAGCACAAGCTCAGGACATGGAGGTGC

***HRAS* v.11** | Exon 1 / Exon 4 / Exon 5 / Exon 6 / Exon 7 | @bac214ad-0aa2-48ee-802f-aff3bd3760ba

GCAGTCGCGCCTGTGAACGGGAGCAGATCAAACGGGTGAAGGACTCCGGATGACGTGCCCATGGTGCTGGTGGGAACAAGTGTGACCTGGCTGCACACTGTGGGGTCTCGGCAGGCTCAGGACCTCGCTGACTGCGGCATCCCTACATCGAGACCTCGGC
CAAGACCCGGCAGGGCAGCCGCTCTGGCTCTAGCTCCAGCTCCAGGACCCTCTGGGACCCCAGGACCCATGTGACCCAGCGGCCCCTCGCGCTGGAGTGGAGGATGCCTTCTACACGTTGGTGCGTCGGCAGCACAAGCTGCGGAAGCTGAACCCTCCTG
ATGAAATTGGCCCCGGCTGCTGAAGGCTGCAAGTGTGTGCTCTCCTGACGCAGCACAAGCTCAGGACATGGAGGTGC

***HRAS* v.12** | Exon 1 / Exon 4 / Exon 5 / Exon 6 (514nt) | @b856cb7f-e1db-4ed6-aa48-636cc4ea1bec

GCAGTCGCGCCTGTGAACGGGAGCAGATCAAACGGGTGAAGGACTCGATGACGTGCCCATGGTGCTGGTGGGGAACAAGTGTGACCTGGCTGCACGCACTGTGGAACTTCGGCAGGCTCAGGACTCGTTCGAAGCTACGGCATCCCCACATCGAGACCTC
GGCCAAGACCCGGCAGGGCAGCCGCTCGCTCGCCCAGCCTGGGACCCCCTCTGACCCCTGGGACCCATGTGACCCAGCGGCCCCTCGCGCTGGAGTGGAGGATGCCTTCTACACGTTATGCGTGAGATCTTCGGCAGCATTGCTGCGGAAGCTGAACCTC
CTGATGAGAGGCCCTGGCTGCATGAGCTGCAAGTGTGTGCTCTCCTGACGCAGGTGAGGGACTCCCAGGGCGGCCGCCACGCCCACCGGATGACCCTGCTCCCCGCCCCTGCCGGTCTTCCTGCCTGCGGTCAGCAGCCTCCCTTGTGCCCCGCCCAGCA
CAAGCTCAGGACATGGAGGTGC

***HRAS* v.13** | Exon 1 / Exon 5 / Exon 6 / Exon 7 | @931d2857-0bd6-472b-aa13-7f82c4eb92f8

GCAGTCGCGCCTGTGAACGGGCAGCCGCTCTGGCTCTAGCTCCAGCTCCGGCAGGGACCTCTCTGTGGACCCCCCGGGACCCATGTGACCCAGCGGCCCCTCGCGCTGGAGTGGAGGATGCCTTCTACGTTGGTGCGTGAGATCCGGCAGCACAAGCTGC
GGAAGCTGAACCCTCCTGATGAGGTGGCCCCGGCTGCATGAGCTGCAAGTGTGTGCTCTCCTGACGCAGCACAAGCTCAGGACATGGAGGTGC

***HRAS* v.14** | Exon 1 / Exon 5 / Exon 6 (514nt) | @5007e468-ecc8-4fbb-9cce-4c96d6664ab7

GCAGTCGCGCCTGTGAACGGGCAGCCGCTCTGGCTTCTAGTCTACCTGGGACCCTCTGACCCCCGACCCATGTGACCCAGCGGCCCCTCGCGCTGGAGTGGAGGAGATGCTCTCTACACGTTGCGCAAGAGATCCGGCAGCACAAGCTGCGGAAGCTGAA
CCTCCTGATGAGAGTGGCCCTGGCTGCATGAGCTTTTGCGTATGCTCTCCTGACGCAGGTGAGGGGACTCCCAGGGCGGCTGCCACGCCTCCACCGGATGACCCGGCTCCCGCCCTGCCGGTCTCCTGGCCTGTGTCAGCAGCTCCCTTGTGCCCCGCCC
AGCAATAAGTCCCAGGACATGGAGGTGC

***HRAS* v.15** | Exon 1 / Exon 6 / Exon 7 | @7ab1e012-0f77-44ee-8997-4defce9f3ed3

GCAGTCGCGCCTGTGAACGGGAGTGGAGGATGCCTTCACACGCTGGTGTGAGATCCGCAGCACAAGCTGCGGAAGCTGAACCCTCCTTGATGAGAGTGGCCCTGGCTGCATGAGCTGCAAGTGTGTGTGCTCTCCTGACGCAGCACAAGCTCAGGACATG
GAGGTGC

***HRAS* v.16** | Exon 1 / Exon 6 (514nt) | @464d7e29-442e-4869-b1ca-ad4d2a2ede6e

GCAGTCGCGCCTGTGAACGGGAGTGGAGGATGCCTTCTACACGTTGGTGCGTGAATCCGGCAGCACAAGCTGCGGAAGCTGAACCCTCCTGATGAGAGTGGCCCCGGCTGCGCAGGCTGCAAGTGTGTGCTCTCCTGACGCAGGTGAGGGGACTCCAGAT
TTGTGCGCCACGCCCACCGGATGACCCCGAAACACCACCCTGCCGGTCTCCTGGCCTGCGGTCAGCGGCCTCCCTTGTGCCCCGCCCAGCACAAGCTCAGGACATGGAGGTGC

***HRAS* v.17** | Exon 1 / Exon 2 / Exon 4 / Exon 6 / Exon 7 | @f0b77cce-5771-4828-ace2-4e8d0b7e67af

GCAGTCGCGCCTGTGAACGGTGGGGCAGGAGACCCTGTAGGGAGGTGGACCTCGGCCGCAGGCCCCTGAGGAGCGATGACGGAATATAAGCTGCGGTGGTGGGCGCCGGCGGTGTGGGGCAAGAGTGCGCTGACCATCCAGCTGATCCAGAACCATTTTG
TGGACGAATACGACCCCCACTATAGAGGGAGCAGATCAAACGGGTGAAGGACTCGGATGACGTGCCCATGGTGCTGGTGTGGGGAACAAGTGTGACCCGGCTGCACGCACTGTGGAATCTCGGCAGGCTCAGGACCTGCTCAGCTACGGCAGCATCCCCT
GGGCATCGAGACCCGGCCAAGACCCGGCAGGGAGTGGAGGATGCCTTCTACACGTTGGTGCAGATCCGGCAGCACAAGCTGCGGAAGCTGAACCCTCCTGATGAGAGTGGCCCCGGCTGCATGAGCTGCAAGTGTGTGCTCTCCTGACGCAGCACAAGCT
CAGGACATGGAGGTGC

***HRAS* v.18** | Exon 1 / Exon 2 / Exon 4 / Exon 6 (514nt) | @e54fb437-a8f3-4150-99b8-f6388fc372f0

GCAGTCGCGCCTGTGAACGGTGGGGCAGGAGACCCTGTAGGATGCACCCCCGGGCCGCAGGCCCCTGAGGAGCGATGACGGAATATAAGCTTGTGTGGTGGTGGGCGCCGGCGGTGGGCAAGAGTGCGCTGAATTCTCATCCAGCCGGATCCAGAACCAT
TTTGTGGACGAATACGACCCCACTATAGAGGGAGCAGATCAACGGGTGAAGGACTCGGATGGCGTGCCCATGGTGCGGTGGGGGAACAACAAGTGTGACCTGGCTAAAATGCACTGTGGAACTCGGCGCCTAGGACTCGCCCGAAGCTACGGCATCCCCA
TCGAGACCTCGGCCAAGACCCGGCAGGGAGTGGAGGATGCCTTCTACACGTTGGTGCGTGAGATCCGGCAGAGCACAAGCTGCGGAAGCTGAACCCTCCTGATGAGTGGCCCCGGCTGCATGATGCGTGTGCTCTCCTGACGCAGGTGAGGGGAATTCCA
GGCTGCCGGCCACGCCCACCGGATGATCTGGCTCTGCCCCTGCCGGTCTCCTGGCCTGCGGTCAGCAGCCTCCCCTTGTGGCCCCGCCCAGCACAAGCTCAGGACATGGAGGTGC

Ion Torrent sequencing reads confirming the novel splice junctions

KRAS Exon 2 / Exon 4 – @0403W:01920:02989

CGTAGGCAAGAGTGCCTTGACGATACAGCTAATTCAGAATCATTTTGTGGACGAATATGATCCAACAATAGAGAGAACAAATTAAAAGAGTTAAGGACTCTGAAGATGTACCTATGGTCCTAGTAGGAAATAAATGTGATTTGCCTTCTAGAACAGTAGA

KRAS Exon 2 / Exon 5 – @0403W:01054:02562

GACGTAGGCAAGAGTGCCTTGACGATACAGCTAATTCAGAATCATTTTGTGGACGAATATGATCCAACAATAGAGAGAGTGGAGGATGCTTTTTATACATTGGTGAGAGAGATCCGACAATACAGATTGAAAAAAATCAGCAAAGAAGAAAAGACTCCTG

KRAS Exon 2 / Exon 6 – @0403W:02065:03307

GTAGGCAAGAGTGCCTTGACGATACAGCTAATTCAGAATCATTTTGTGGACGAATATGATCCAACAATAGAGGGTGTTGATGATGCCTTCTATGTAGGAAATAAATGTGATTTGCCTTCTAGAACAGTAGACACAAAACAGGCTCAGGACTTAGCAAGAA

KRAS Exon 1 (179 bp) / Exon 3 – @0403W:01910:02711

GGACTGGGAGCGAGCGCGGCGCAGGCACTGAAGGCGGCGGCGGGGCCAGAGGCTCAGCGGCTCCCAGGTGCGGGAGAGAGGATTCCTACAGGAAGCAAGTAGTAATTGATGGAGAAACCTGTCTCTTGGATATTCTCGACACAGCAGGTCAAGAGGAGTA

KRAS Exon 1 (166 bp) / Exon 3 – @0403W:01975:00921

CTGGGAGCGAGCGCGGCGCAGGCACTGAAGGCGGCGGCGGGGCCAGAGGCTCAGCGGCTCCCAGGATTCCTACAGGAAGCAAGTAGTAATTGATGGAGAAACCTGTCTCTTGGATATTCTCGACACAGCAGGTCAAGAGGAGTACAGTGCAATGAGGGAC

KRAS Exon 1 (179 bp) / Exon 4 – @0403W:01507:03156

GGAGCGAGCGCGGCGCAGGCACTGAAGGCGGCGGCGGGGCCAGAGGCTCAGCGGCTCCCAGGTGCGGGAGAGAGAGAACAAATTAAAAGAGTTAAGGACTCTGAAGATGTACCTATGGTCCTAGTAGGAAATAAATGTGATTTGCCTTCTAGAACAGTAG

KRAS Exon 1 (166 bp) / Exon 4 – @0403W:01785:02091

GGAGCGAGCGCGGGCGCAGGCACTGAAGGGCGGCGGCGGGGCCAGAGGGCTCAGCGGCTCCCAGAGAACAAATTAAAAGAGTTAAGGACTCTGAAGATGTACCTATGGTCCTAGTAGGAAATAAATGTGATTTGCCTTCTAGAACAGTAGACACAAAACA

KRAS Exon 1 (179 bp) / Exon 5 – @0403W:01009:02159

GCGGCGCAGGCACTGAAGGCGGCGGCGGGGCCAGAGGCTCAGCGGCTCCCAGGTGCGGGAGAGAGAGAGTGGAGGATGCTTTTTTATACATTGGTGAGAGAGATCCGACAATACAGATTGAAAAAAAAATCAGCAAAGAAGAAAAAGACTCCTGGCTGTG

KRAS Exon 1 (179 bp) / Exon 6 – @0403W:00792:01291

AAGGCGGCGGCGGGGCCAGAGGCTCAGCGGCTCCCAGGTGCGGGAGAGAGGGTGTTGATGATGCCTTCTATACATTAGTTCGAGACTAAATCATTTGAAGATATTCACCATTATAGAGAACAAATTAAAAGAGTTAAGGACTCTGAAGATGTACCTATGG

NRAS Exon 2 / Exon 4 – @0403W:01961:03127

GAAAAGCGCACTGACAATCCAGCTAATCCAGAACCACTTTGTAGATGAATATGATCCCACCATAGAGGGAGCAGATTAAGCGAGTAAAAGACTCGGATGATGTACCTATGGTGCTAGTGGGAAACAAGTGTGATTTGCCAACAAGGACAGTTGATACAAA

NRAS Exon 2 / Exon 5 – @0403W:01861:02332

TGGGAAAAGCGCACTGACAATCCAGCTAATCCAGAACCACTTTGTAGATGAATATGATCCCACCATAGAGGGTGTTGAAGATGCTTTTTACACACTGGTAAGAGAAATACGCCAGTACCGAATGAAAAAACTCAACAGCAGTGATGATGGGACTCAGGGT

NRAS Exon 3 / Exon 5 – @0403W:01029:01339

GACAGGCGAAGGCTTCCTCTGTGTATTTGCCATCAATAATAGCAAGTCATTTGCGGATATTAACCTCTACAGGGTGTTGAAGATGCTTTTTACACACTGGTAAGAGAAAATACGCCAGTACCGAATGAAAAAACTCAACAGCAGTGATGATGGGACTCAG

NRAS Exon 5 / Exon 7 – @0403W:01133:01036

GAAATACGCCAGTACCGAATGAAAAAACTCAACAGCAGTGATGATGGGACTCAGGGTTGTATGGGATTGCCATGTGTGGTGATGTAACAAGCTGCACTGACACCCTGGTCCTGACTTCCCTGGAGGAGAAGTATTCCTGTTGCTGTCTTCAGTCTCACAG

NRAS Exon 1 / Exon 3 – @0403W:00169:02151

GCTGTGGTCCTAAATCTGTCCAAAGCAGAGGCAGTGGAGCTTGAGGATTCTTACAGAAAACAAGTGGTTATAGATGGTGAAACCTGTTTGTTGGACATACTGGATACAGCTGGACAAGAAGAG

NRAS Exon 1 / Exon 4 – @0403W:01169:02653

GCTGTGGTCCTAAATCTGTCCAAAGCAGAGGCAGTGGAGCTTGAGGGAGCAGATTAAGCGAGTAAAAGACTCGGATGATGTACCTATGGTGCTAGTGGGAAACAAGTGTGATTTGCCAACAAGGACAGTTGATACAA

NRAS Exon 1 / Exon 5 – @0403W:01457:02283

GCTGTGGTCCTAAATCTGTCCAAAGCAGAGGCAGTGGAGCTTGAGGGTGTTGAAGATGCTTTTTACACACTGGTAAGAGAAATACGCCAGTACCGAATGAAAAAACTCAACAGCAGTGATGATGGGACTCAGGGTTGTATGGGATTGCCATGTGTGGTGA

HRAS Exon 1 / Exon 3 – @FS82K:01967:00841

CGCAGTCGCGCCTGTGAACGGATTCCTACCGGAAGCAGGTGGTCATTGATGGGGAGACGTGCCTGGTTGGACATCCTGGATACCGCCGGCCAGGAGGAGTACAGCGGCCATGCGG

HRAS Exon 1 / Exon 4 – @FS82K:01459:01908

GATGCAGTCGCGCCTGTGAACGGGAGCAGATCAAACGGGTGAAGGACTCGGATGACGTGCCCATGGTGCTGGTGGGGAACAAGTGTGACCTGGCTGCACGCACTGTGGAATCTCGGCAG

HRAS Exon 1 / Exon 5 – @FS82K:01421:01779

CGGGGGCAGTCGCGCCTGTGAACGGGCAGCCGCTCTGGCTCTAGCTCCAGCTCCGGGACCCTCTGGGACCCCCCGGGAC

HRAS Exon 1 / Exon 6 / Exon 7 – @FS82K:01029:01993

GCCTGTGAACGGGAGTGGAGGATGCCTTCTACACGTTGGTGCGTGAGATCCGGCAGCACAAGCTGCGGAAGCTGAACCCTCCTGATGAGAGTGGCCCCGGCTGCATGAGCTGCAAGTGTGTGCTCTCCTGACGCAGCACAAGCTCAGGACATGGAGGTGC

HRAS Exon 2 / Exon 4 – @FS82K:01703:00631

GTGGTGGGCGCCGGCGGTGTGGGCAAGAGTGCGCTGACCATCCAGCTGATCCAGAACCATTTTGTGGACGAATACGACCCCACTATAGAGGGAGCAGATCAAACGGGTGAAGGACTCGGATGACGTGCCCATGGTGCTGGTGGGGAACAAGTGTGACCTG
