# Supplementary material for: Targeted Long-Read Sequencing Decodes the Transcriptional Atlas of the Founding RAS Gene Family Members
Source: Int J Mol Sci. 2021 Dec 10;22(24):13298. doi: 10.3390/ijms222413298 (PMC8709048; doi:10.3390/ijms222413298)
Supplement: Supplementary file 1 [file ijms-22-13298-s001.zip › Table S1.pdf]

Table S1. List of the human cell lines that were propagated for the present work. All cell lines were cultured based on the American Type Culture Collection (ATCC) guidelines.

No	Human tissue/type of malignancy	Human Cell lines
1	Breast cancer	MCF-7, BT-20, SK-BR-3, MDA-MB-231, MDA-MB-468, BT-474
2	Ovarian cancer	ES-2, SK-OV-3, OVCAR-3, MDAH-2774
3	Prostate cancer	PC-3, DU 145, LNCaP
4	Colorectal cancer	DLD-1, HT-29, Caco-2, HCT 116, SW620
5	Urinary bladder cancer	RT4, T24
6	Liver cancer	Hep G2, HuH-7
7	Lung cancer	A549
8	Cervical cancer	HeLa, SiHa
9	Brain cancer	SH-SY5Y, H4, U-87 MG, U-251 MG
10	Haematological malignancies	Raji, SU-DHL-1, HL-60
11	Gastric cancer	AGS
12	Endometrial cancer	Ishikawa, SK-UT-1B
13	Renal cell cancer	786-O, Caki-1
14	Non-cancerous cell lines	HEK-293, HaCaT, BJ
